# Doppler ultrasound findings correlate with tissue vascularity and inflammation in surgical pathology specimens from patients with small intestinal Crohn’s disease

**DOI:** 10.1186/1756-0500-7-363

**Published:** 2014-06-14

**Authors:** Tomohiko Sasaki, Reiko Kunisaki, Hiroto Kinoshita, Hideaki Kimura, Teruaki Kodera, Akinori Nozawa, Akiho Hanzawa, Naomi Shibata, Hiromi Yonezawa, Eiji Miyajima, Satoshi Morita, Shoichi Fujii, Kazushi Numata, Katsuaki Tanaka, Masanori Tanaka, Shin Maeda

**Affiliations:** 1 Gastroenterological Center, Yokohama City University Medical Center, Yokohama, Japan; 2 Inflammatory Bowel Disease Center, Yokohama City University Medical Center, Yokohama, Japan; 3 Department of Pathology, Yokohama City University Medical Center, Yokohama, Japan; 4 Department of Laboratory Medicine and Clinical Investigation, Yokohama City University Medical Center, Yokohama, Japan; 5 Department of Biostatistics and Epidemiology, Graduate School of Medicine, Yokohama City University, Yokohama, Japan; 6 Department of Surgery, NTT Medical Center Tokyo, Tokyo, Japan; 7 Department of Pathology and Laboratory Medicine, Hirosaki City Hospital, Aomori, Japan; 8 Department of Gastroenterology, Yokohama City University Graduate School of Medicine, Yokohama, Japan

**Keywords:** Crohn’s disease, Small intestine, Color doppler ultrasound, Vascularity, Inflammation, Fibrosis

## Abstract

**Background:**

Crohn’s disease (CD) is routinely evaluated using clinical symptoms, laboratory variables, and the CD activity index (CDAI). However, clinical parameters are often nonspecific and do not precisely reflect the actual activity of CD small-intestinal lesions. The purposes of this prospective study were to compare color Doppler ultrasound (US) findings with histological findings from surgically resected specimens and confirm the hypothesis that color Doppler US can distinguish tissue inflammation and fibrosis.

**Methods:**

Among 1764 consecutive patients who underwent color Doppler US examinations, 10 patients with CD (12 small-intestinal CD lesions) who underwent US examinations before elective small-intestine resection were evaluated in the present study. Areas of thickened intestinal walls were evaluated in terms of blood flow using color Doppler US imaging. The blood flow was semiquantitatively classified as “hyper-flow” and “hypo-flow” according to the Limberg score. Resected lesions were macroscopically and histopathologically processed. Inflammatory cell infiltration, fibrosis and vascularity were evaluated by myeloperoxidase (granulocytes), CD163 (macrophages), CD79a (B cells), CD3 (T cells), Masson’s trichrome (fibrosis), and factor VIII staining (vascular walls). All histopathological images were entered into virtual slide equipment and quantified using a quantitative microscopy integrated system (TissueMorph™).

**Results:**

There were no significant differences in disease features or laboratory findings between “hypo-flow” lesions (n = 4) and “hyper-flow” lesions (n = 8). Histopathologically, “hyper-flow” lesions showed significantly greater bowel wall vascularity (factor VIII) (*p* = 0.047) and inflammatory cell infiltration, including CD163 macrophages (p = 0.008), CD3 T cells, and CD79a B cells (*p* = 0.043), than did “hypo-flow” lesions. There was no apparent association between the blood flow and CDAI.

**Conclusions:**

In this study, active CD lesions were macroscopically visible in surgical specimens of patients with increased blood flow on preoperative color Doppler US imaging. Additionally, these CD lesions exhibited significantly greater vascularity and numbers of inflammatory leukocytes microscopically. Color Doppler US may predict tissue inflammation and fibrosis in small-intenstinal CD lesions.

## Background

Crohn’s disease (CD) is a chronic inflammatory disease of the intestine that follows a pattern of relapse and remission characterized by segmental and transmural inflammation that can lead to luminal stricture. CD activity is generally assessed using a combination of clinical symptoms, blood tests, the CD activity index (CDAI), and imaging techniques [1,2]. However, clinical symptoms and laboratory test results are often nonspecific, and judging whether these signify the presence of active lesions or obstruction due to fibrotic strictures is generally difficult. Especially, a lesion in the small intestine lying deep in the abdomen rarely contributes to clinical symptoms or is detected in blood tests, even in patients with relapsing enteritis, and is difficult to approach by endoscopy. Numerous studies have demonstrated the ability of a variety of imaging modalities to predict CD disease activity [3-9], but to date, no study has been able to assess the degree of inflammation accurately. Because a single absolute reference method to assess disease activity is lacking, multiple parameters and imaging modalities are often used.

The European guidelines for CD diagnosis recommend transabdominal ultrasound (US), magnetic resonance imaging (MRI), and computed tomography (CT) enterography/enteroclysis as minimally invasive alternatives to endoscopy and contrast imaging for examination of the small intestine [10-12]. Color Doppler US is a noninvasive method for evaluating intestinal wall blood flow; therefore, this method should be appropriate for assessment of CD activity [13,14]. When compared with conventional CT or MRI, transabdominal US has the advantage of high spatial resolution, which allows external examination of the five-layered mural structure and transmural hypervascularity in the inflamed intestinal wall, and may help to detect the inflammatory activity of focal CD lesions [15-18].

We investigated whether or not increased blood flow to the intestinal wall observed on US implies histopathological inflammation of CD lesions. Data on histological correlations of color Doppler US are still lacking, and few studies have performed histological evaluations of a small number of cases [19-21]. Accordingly, we aimed to compare intestinal wall blood flow by color Doppler US with quantitatively measured histopathological findings of inflammatory cell infiltration and vascularity in resected CD small-intestinal lesions.

## Methods

### Patient recruitment

Patients who underwent color Doppler US examinations at the Yokohama City University Medical Center from January 2008 to March 2012 were identified. Patients with CD who underwent preoperative transabdominal US prior to surgical resection of small-intestinal lesions were eligible for this study. To ensure that the lesion observed with color Doppler US was the same as that surgically resected, patients with a single lesion (or if multiple lesions were present, those close to the ileocecal valve) were included. Patients with internal or external fistulae, abscesses, and severe obstruction were excluded to eliminate the possible histological influences of secondary infection such as intestinal obstruction or abscess formation. Consequently, macroscopically representative CD cases were included, such as those with longitudinal ulcers, a cobblestone appearance, and fibrotic stenosis.

Prior to surgery, a detailed clinical history was collected, including age, sex, CD duration, previous segmental resection, disease location, disease behavior, preoperative therapy, site and duration of disease, number of clinical recurrences, and type of previous medical therapy. All patients were evaluated for clinical and biochemical activity in terms of CDAI, C-reactive protein, and erythrocyte sedimentation rate. Patients were excluded if the clinical data were incomplete.

### Transabdominal US examinations

The examinations were performed by three highly experienced technicians (H.Y., N.S., and A.H.) together with a gastroenterologist (T.S.). The US examinations were performed without any special preparation; no prior fast, parenteral contrast reagents, or administration of oral contrast media to achieve adequate distension. The US equipment used was Aplio XG and XV (Toshiba Medical Systems Corp., Tochigi, Japan). A general scan was performed using a 6-MHz convex probe, and a more localized and detailed scan of the affected area was conducted using a 7.5-MHz linear probe. In the examination, the terminal ileum was identified using the ileocecal valve, after which the ileum was followed rostrally as continuously as possible for evaluation. When the examiners lost track of the intestine continuity, they identified a nearby intestine from which they resumed the examination, and the entire abdomen was searched for any detectable part of the small intestine using the graded compression technique [22].

**Figure 1:**
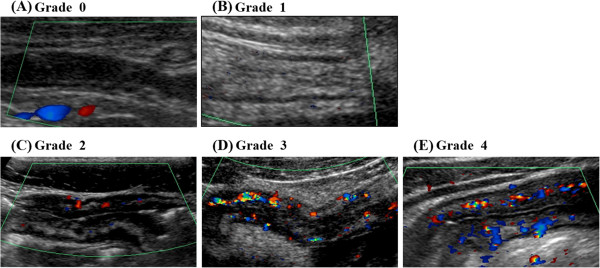
**Examples of semiquantitative assessment of bowel wall vascularity using Doppler US according to Limberg score. (A)** Grade 0 = no bowel wall thickening, no vascularization; **(B)** Grade 1 = bowel wall thickening without vascularization; **(C)** Grade 2 (hypo-flow) = bowel wall thickening with short stretches of vascularity as spots; **(D)** Grade 3 (hyper-flow) = bowel wall thickening with longer stretches of vascularity; **(E)** Grade 4 (hyper-flow) = bowel wall thickening with longer stretches of vascularity reaching the mesentery.

Areas with thicker walls than the surrounding intestine were considered to be pathologically affected. All thickened areas of the intestine were evaluated in terms of blood flow using color Doppler US imaging. The range of blood flow velocities for the area of interest was adjusted to 4 cm/sec, and the color gain was reduced from the maximum value and evaluated at the maximum gain that eliminated noise. The blood flow was then evaluated using the Limberg score (Figure 1) [19,23] and macroscopically and semiquantitatively graded from Grade 0 to 4. Grade 0 was defined as a normal intestinal wall without a color Doppler signal, without bowel wall thickness, and with a preserved five-layer wall structure. Grade 1 was defined as hypoechoic intestinal wall thickness with partially obliterated wall layers but without increased vascularity (no color Doppler signal). Grade 2 was defined as intestinal wall thickness with short stretches of vascularity (spot). Grade 3 was defined as longer stretches of vascularity, and Grade 4 as longer stretches of vascularity extending into the surrounding mesentery.

In this study, we classified Grade 1 cases as “hypo-flow” and Grade 3 and 4 cases as “hyper-flow.” Grade 0 was excluded because it was not a CD lesion and was considered normal. Grade 2 was excluded because it was intermediate and not a representative image and was thus considered unsuitable for this analysis.

As described above, all cases that did not match the preoperative US images and postoperative surgical specimens were excluded from the study. Other excluded cases were patients with deep lesions that did not accommodate proper flow evaluation and patients with gas retention due to obstruction, which made bowel evaluation difficult.

### Surgery

The surgical treatments for the CD lesions were performed by an experienced surgeon specialized in inflammatory bowel disease (IBD). The resected area with the lesion was opened longitudinally from the side opposite to the mesenteric attachment and then fixed in buffered formalin. The pathology slide was prepared with transverse slices made at the site of pathological lesions observed on the US examination.

### Histopathological analyses

Resected small-intestinal segments were retrieved and evaluated. When the resected segment for pathological examination was prepared, the lesion location had to match exactly with color Doppler US findings. Thus, we selected the single lesion, or if there were several lesions, the existing terminal ileum. If the co-identity of the lesion in the surgical and US findings was questionable, the case was excluded. The specimen was transversely divided through the approximate lesion center and subjected to histological examination.

**Figure 2:**
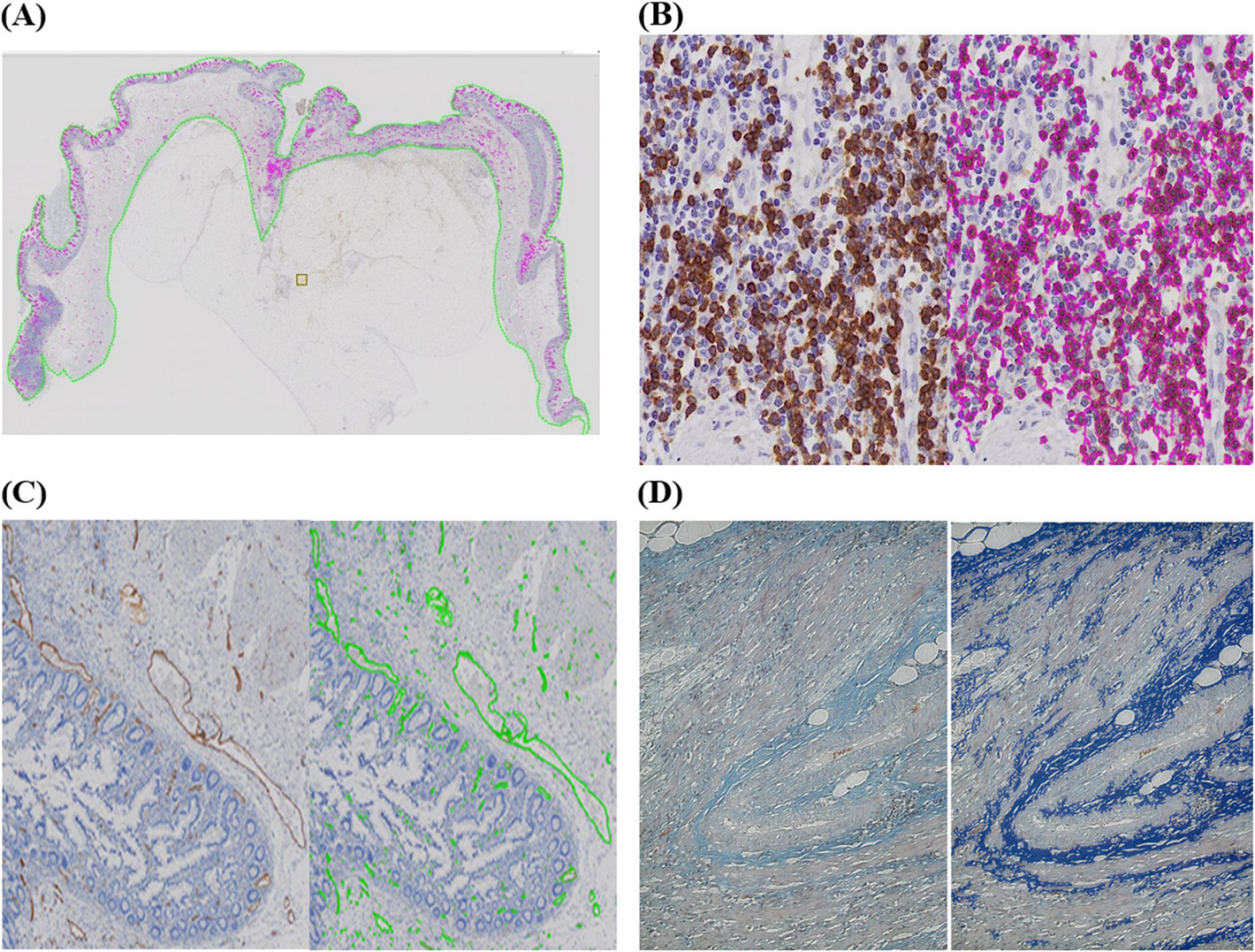
**Histopathological images quantified using an automated quantitative microscopy integrated system (TissueMorph™ by VisioPharm, Hoersholm, Denmark). (A)** Immunohistochemical staining area for the entire cross-sectional analysis was recognized and delineated by TissueMorph™. **(B)** Determination of positive staining for hematopoietic cells. Immunological staining of cell-membrane markers (left panel) and analysis results (right panel). **(C)** Evaluation of vasculature. Immunological staining of the vascular walls using factor VIII (left panel) and analysis results (right panel). **(D)** Determination of fibrosis. The faint blue color of positive Masson trichrome staining (left panel) was enhanced (right panel) and identified for measurement.

The overall image of the selected sample was evaluated after hematoxylin and eosin (H&E) staining. All samples were reviewed by an independent expert gastrointestinal pathologist (T.M.) and excluded if tissue specimens were considered unsuitable for study. Evaluation of inflammatory cell infiltration was performed by myeloperoxidase (MPO) staining for granulocytes (Dako, Glostrup, Denmark), CD163 staining (Leica MICROSYSTEMS, Wetzlar, Germany) for macrophages, CD79a staining (Roch Diagnostics GmbH, Mannheim, Germany) for B cells, and CD3 staining (Roch Diagnostics GmbH, Mannheim, Germany) for T cells. Masson trichrome staining was used to evaluate fibrosis, and factor VIII staining (Dako, Glostrup, Denmark) was used to identify vascular walls.Digital images were created from all slides using a whole slide scanner (OliVIA VS120: Olympus Corp., Tokyo, Japan). The resulting images were digitized and quantified using an automated quantitative microscopy integrated system (TissueMorph™: VisioPharm, Hoersholm, Denmark). TissueMorph™ is an analysis software module based on digitized glass slides; it enables cross-sectional quantitative analysis of bowel-wall tissue properties, excluding the mesenteric fat (Figure 2A). The cell membranes (Figure 2B) and vascular endothelium (Figure 2C) were stained using an immunologic staining method, and fibrotic areas stained using Masson trichrome (Figure 2D) were evaluated and the corresponding area was quantitated. The sum of this calculation was divided by the area used for the pathological analysis, and the values per unit area were multiplied by 10,000 for conversion into more practical figures.

Specimens from three patients in whom a right hemicolectomy was performed during the same period due to colon cancer were used as controls *versus* surgical specimens.

### Ethical considerations

Before contacting the study subjects, our protocol was reviewed and approved by the Screening Committee of the Yokohama City University Medical Center. Likewise, informed consent was obtained from all 10 patients who were eligible and agreed to participate in this study. Additionally, the Principle of Good Clinical Practice and the Declaration of Helsinki were adhered to at all times.

### Statistics

The IBM SPSS Statistics software, version 19 (IBM Japan, Ltd., Tokyo, Japan) was used for statistical analysis. For comparison of continuous variables from two data sets, the *t*-test was applied. Due to the small sample size, Fisher’s exact test was used to compare data sets, and a value of *p* < 0.05 was considered to indicate statistical significance.

## Results and discussion

### Baseline preoperative transabdominal color Doppler imaging

**Figure 3:**
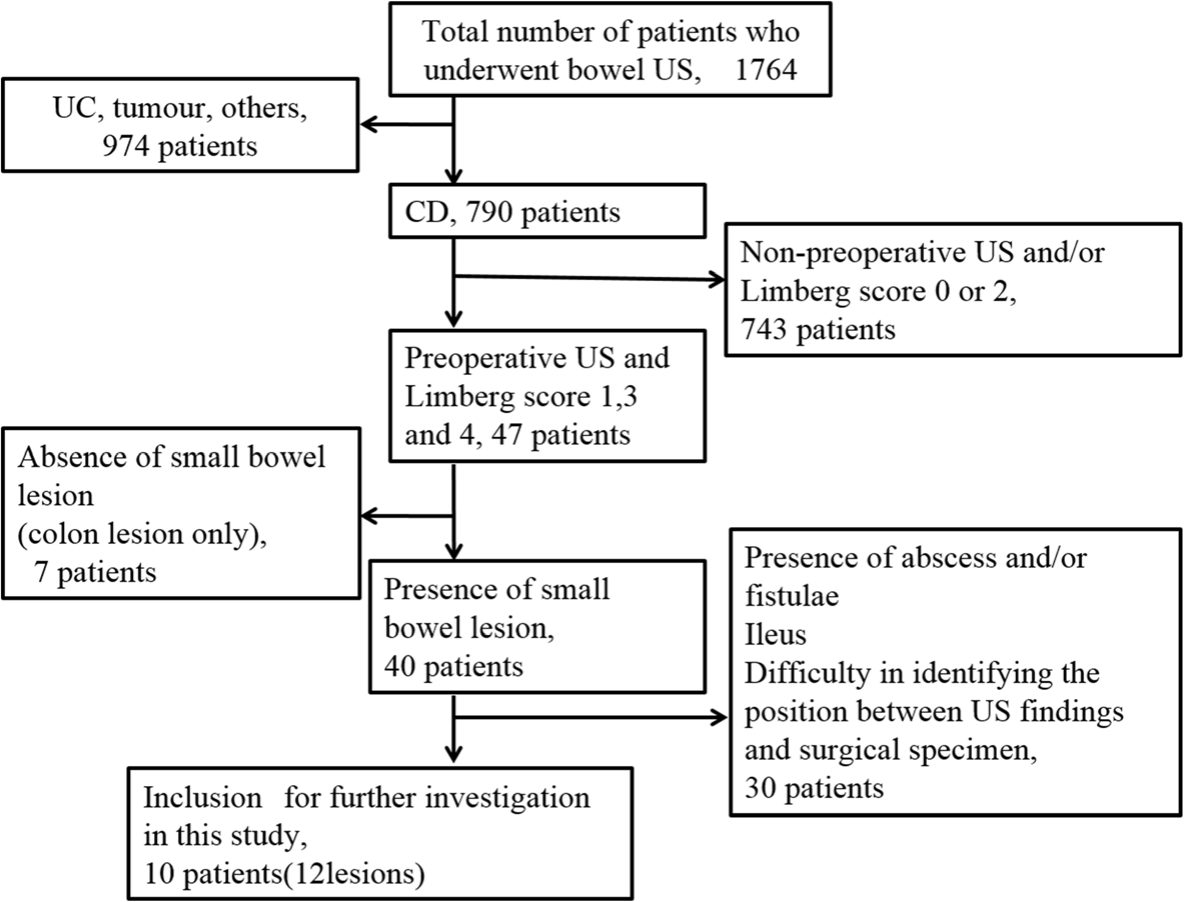
**Inclusion and exclusion criteria for this study.** A total of 1,764 patients underwent color Doppler US examinations from January 2008 to March 2012; of these, 790 patients had CD. In total, 743 patients who did not undergo US before the operation and/or whose Limberg score of Doppler US was 0 or 2 were excluded. Forty-seven patients underwent surgery and had Grade 1, 3, or 4 Limberg score. Seven patients without small bowel lesions (colon lesions only) were excluded, and 30 patients with abscesses, fistulae, ileus, and/or difficulty in identifying the position between US findings and surgical specimens were also excluded.

A total of 1,764 patients underwent color Doppler US examinations from January 2008 to March 2012; of these, 790 patients had CD, and 743 patients who did not undergo US before the operation and/or whose Limberg score of Doppler US was 0 or 2 were excluded. Forty-seven patients underwent surgery and had a Grade 1, 3, or 4 Limberg score. Seven patients without small bowel lesions (colon lesions only) were excluded, and 30 patients with abscesses, fistulae, ileus, and/or difficulty in identifying the position between US findings and surgical specimens were also excluded (Figure 3). The mean interval between surgery and US examinations was 6 days (range, 1–16 days). Thirty patients were excluded based on our criteria. The clinical and biochemical characteristics of 12 lesions in 10 patients fulfilled the inclusion criteria and were considered for analysis (Table 1). The most common indication for surgery was recurrent obstruction associated with lesions in the small intestine.

**Table 1:** Baseline demographic variables, disease features, and laboratory findings in patients with Crohn’s disease (CD) selected for color Doppler ultrasound evaluations of CD lesions in the small intestine

**Demography**	**“Hypo-flow”**	**“Hyper-flow”**	***p *** **value**
	(n = 4)	(n = 8)	
Age in years	40.0 ± 8.2	26.8 ± 13.8	0.111^1)^
Male (%)	2 (50.0)	3 (37.5)	1.000^2)^
CD duration in years	13.8 ± 7.8	3.9 ± 4.2	0.081^1)^
Previous segmental resection, %	0	0	
Disease location, n (%)*			0.491^2)^
L1 (distal 1/3 ileum ± limited cecal)	0 (0.0)	3 (37.5)	
L3 (ileocolonic)	4 (100.0)	5 (62.5)	
L4b (upper, distal to ligament of Treitz and proximal to distal 1/3 ileum)	0 (0.0)	0 (0.0)	
Disease behavior, n (%)*			
B1 (nonstricturing, nonpenetrating)	0 (0.0)	0 (0.0)	
B2 (stricturing)	4 (100.0)	8 (100.0)	
B3 (penetrating)	0 (0.0)	0 (0.0)	
B4 (structuring and penetrating)	0 (0.0)	0 (0.0)	
Primary indication for surgery, n (%)			1.000^2)^
Recurrence of obstruction symptoms	4 (100.0)	7 (87.5)	
Intractable disease	0 (0.0)	1 (12.5)	
CDAI	196.2 ± 73.8	198.2 ± 63.9	0.963^1)^
C reactive protein (mg/dl)	1.30 ± 2.01	0.47 ± 0.34	0.466^1)^
Erythorocyte sedimentation rate (mm/h)	32.0 ± 30.0	16.9 ± 14.3	0.251^1)^
Preoperative therapy, n (%)			
5ASA	4 (100.0)	5 (62.5)	0.491^2)^
Elemental diet (>900 kcal)	4 (100.0)	7 (87.5)	1.000^2)^
TPN	2 (50.0)	3 (37.5)	1.000^2)^
CS (prednisolone)	0 (0.0)	4 (50.0)	0.208^2)^
AZA/6MP	0 (0.0)	4 (50.0)	0.208^2)^
Anti-TNF-α biologics	1 (25.0)	0 (0.0)	0.333^2)^
Antibiotics	2 (50.0)	3 (37.5)	1.000^2)^

^1)^Student’s t-test; ^2)^Fisher’s exact test; *Paris classification of CD; CDAI, Crohn’s disease activity index (≥150 indicates active disease); 5ASA, 5-aminosalycilic acid; 6MP, 6-mercaptopurine; AZA, azathioprine; TPN, total parenteral nutrition.

Based on preoperative color Doppler imaging, the cases were classified into “hypo-flow” (n = 4) and “hyper-flow” (n = 8) groups. There was no significant difference between the two groups in disease features and laboratory findings; *e.g.,* disease type and preoperative treatment. Additionally, there was no significant difference in the CDAI of the two groups (Table 1).

### Comparison of preoperative US flow and microscopic quantitative evaluation of inflammatory infiltration, vascularity, and fibrosis

**Figure 4:**
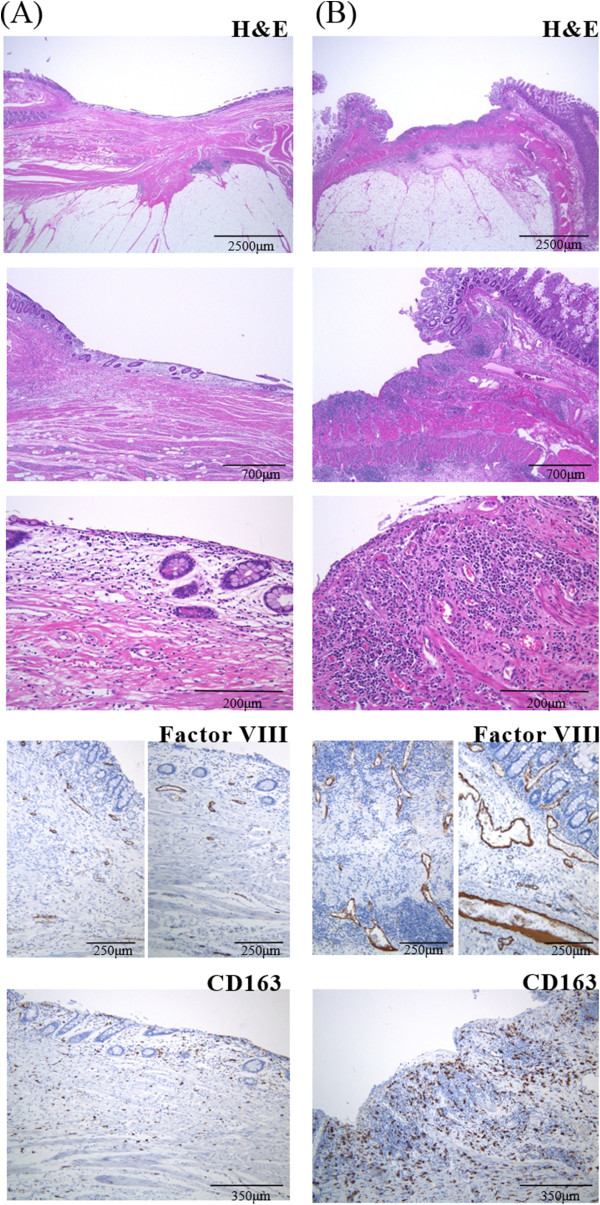
**Representative examples of pathological findings of the (A) “hypo-flow” and (B) “hyper-flow” surgical specimens.** H&E staining revealed more active inflammation in “hyper-flow” **(B)** than in “hypo-flow” **(A)** cases. Vascular walls stained immunohistochemically using an anti-factor VIII antibody and macrophages stained with an anti-CD163 antibody were more prominent in “hyper-flow” **(B)** compared with “hypo-flow” **(A)** cases.

**Figure 5:**
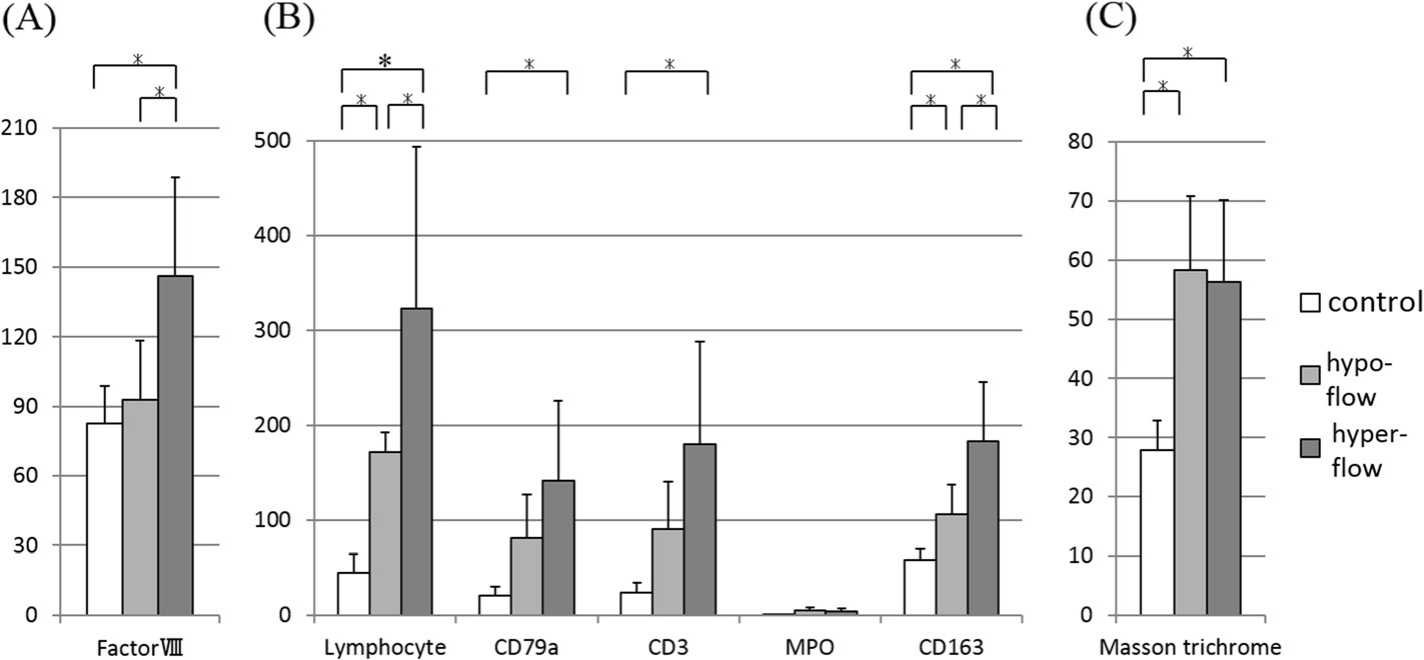
**Histopathological analysis of vascularity, inflammatory cell infiltration, and fibrosis.** Tissue sections of “hyper-flow”, “hypo-flow”, and controls were stained with anti-factor VIII antibody, anti-CD163, CD3, CD79a MPO, or Masson’s trichrome. Positive-staining cells or areas were calculated using TissueMorph™. White, gray, and black bars represent control, “hypo-flow”, and “hyper-flow”, respectively. *p < 0.05. **(A)** The vascular walls were stained using an anti-factor VIII antibody, and the numbers of factor VIII-positive cells were calculated. **(B)** Inflammatory cell infiltrations were stained using anti-CD163, CD3, CD79a, and MPO antibody, and the numbers of positive-staining cells were calculated. **(C)** Fibrotic areas stained using Masson’s trichrome were evaluated, and the corresponding area was quantitated.

Next, we compared the preoperative US flow and histological findings of the resected tissues. H&E stains showed that inflammatory cell infiltration was more severe in the “hyper-flow” group than the “hypo-flow” group (Figure 4). To evaluate inflammation quantitatively, we used recently developed quantitative digital software combined with a whole slide scanner and an automated quantitative microscopy integrated system for immunohistochemical analysis. We found that the numbers of CD163-positive macrophages (*p* = 0.044) and lymphocytes (CD3-positive T cells and CD79a-positive B cells) (*p* = 0.043) were higher in the “hyper-flow” group than in the “hypo-flow” group (Figure 5B). The numbers of CD163-positive macrophages and lymphocytes in both the “hyper-flow” and “hypo-flow” groups was significantly higher than in the control specimens. In contrast, the numbers of MPO-positive granulocytes were almost identical among the groups, including the controls (Figure 5B).

We also performed a vascularity evaluation by immunohistochemical staining using an anti-factor VIII antibody (Figure 4). Vascular length was significantly greater in the “hyper-flow” group than in the control and “hypo-flow” groups (“hyper-flow” *vs.* control, *p* = 0.038; “hyper-flow” *vs.* “hypo-flow,” *p* = 0.047) (Figure 5A). Fibrosis was evaluated by quantitative analysis of Masson trichrome staining. The level of positive Masson trichrome staining in both the “hyper-flow” and “hypo-flow” groups was significantly higher than that in the control specimens (“hyper-flow” *vs.* control, *p* = 0.008; “hypo-flow” *vs.* control, *p* = 0.011), although there was no significant difference between the “hyper-flow” and “hypo-flow” groups (Figure 5C).

In this study, we used a novel quantitative digital software package combined with a whole slide scanner and an automated quantitative microscopy integrated system to evaluate the vascularity and inflammation of thickened small intestinal areas that corresponded to the areas scanned by color Doppler US. In the surgical specimens that showed increased blood flow on the color Doppler US, significant histopathological increases in vascularity and inflammatory cell infiltration were identified. Therefore, color Doppler US is capable of characterizing the inflammatory activity of CD small-intestinal lesions.

Doppler US has become widely used to assess the microvasculature of various organs, and recent findings indicate that it can assess the hemodynamics and inflammatory activity of certain lesions [24-26]. In rheumatoid arthritis, as demonstrated and described in the guidelines [27], there was a strong correlation between inflammation in the affected joints and angiogenesis. In addition, mediators such as fibroblast growth factor, vascular endothelial growth factor, TNF-α, and interleukin-1 promote vascular proliferation in the affected synovial membrane [28-31]. Regarding CD, several reports state that increased blood flow revealed by US may reflect CD disease activity [8,19,32-34]. While few previous reports showed that Doppler US findings correlate with inflammation based on endoscopic biopsy specimens and/or surgical specimens [19-21,32], our study is unique because we used surgical pathology as the gold standard for color Doppler US. Additionally, we investigated the histological inflammation and vascularity of the resected specimen’s intestinal wall and the correlation with the color Doppler US findings using a recently created digital quantitative technique that enables the evaluation of the inflammation, vascularity, and fibrosis of the entire cross-section of the intestinal wall. To our knowledge, this is the first study to investigate the inflammatory cell infiltration and vascularity in all layers of the small-intestinal wall.

Vascular lesions and microvascular changes, such as granulomatous vasculitis, neovascularization, and dilatation of arteries and veins, are well known to be features of the pathogenesis of CD. According to recent reports [35,36], there is new understanding regarding the induction of vascular proliferation during chronic CD inflammation, although there is no general consensus regarding its mechanism. Clinically, we often experience serious bleeding incidents from active CD lesions showing “hyper-flow” according to color Doppler US; such lesions are rapidly and successfully stabilized by administration of anti-TNF-α antibodies. Therefore, color Doppler US may help to determine when anti-TNF-α antibody should be administered for active small-intestinal CD lesions.

Previous reports often described a correlation between the US images of CD lesions and CDAI [8,33,37]. However, in this study there was no significant difference between the CDAI of “hypo-flow” and “hyper-flow” cases (Table 1). Additionally, there was no correlation between CDAI and histological vascularity (factor VIII stain) as analyzed by TissueMorph™ (data not shown). A poor correlation exists between the disease activity of affected small-intestinal lesions and the clinical level of severity as represented by inflammatory markers or the CDAI [38].

Our study has several limitations. It was limited by the small sample size, which included only patients who underwent surgery at a tertiary referral center. To definitively eliminate the possible histological influence of another pathologic process of CD itself, only representative CD lesions were included based on the prespecified inclusion criteria. Because of recent improvements in medical therapy and endoscopic dilatation, most surgical cases during our study were for more complicated conditions, such as internal fistulae or abscess formation, and therefore, fewer cases were available for inclusion. Another reason for the small sample size was the limitation to patients with a Limberg score of 1, 3, or 4 to allow better evaluation of blood flow. Therefore, patients with a Limberg score of 2, which is the most common score, were excluded. In our another study, when we compared the Limberg score of color Doppler US with the endoscopic activity score (simple endoscopic score for CD [SES-CD]), a substantial correlation was identified (ρ = 0.709, *p* < 0.001). A Grade 2 Limberg score was an intermediate grade between “hypo-flow” (Grade 1) and “hyper-flow” (Grade 3 and 4) [39]. Additionally, there was concern regarding the accuracy of transabdominal US; this method has the disadvantages of being dependent on factors such as the experience level of the examining physician [40] and the fact that it is difficult to perform on overweight patients.

## Conclusions

Our investigation outcomes clearly reflected histological CD activity; significant differences were noted in both blood flow and inflammatory cell infiltration between the “hyper-flow” and “hypo-flow” groups, and between the aforementioned and the control specimens. In conclusion, our data suggest that color Doppler US can predict tissue inflammation in CD small-intestinal lesions and is a promising modality for investigation and therapeutic management of CD small-intestinal lesions.

### Availability of supporting data

The data sets supporting the results of this article are included within the article and its additional files.

## Competing interests

All authors declare that they have no competing interests.

## Authors’ contribution

All authors have made significant contributions to this work as specified below. TS, RK, EM, SM, KT and SM: Conception, study design, and preparation of the manuscript draft; RK, HK, HK: Patient management and data generation, collection, assembly, analysis, and interpretation; HK and F: Performance of surgery and analysis and interpretation of data; AH, NS, HY, and KN: Study conception and design, preparation of the manuscript draft, and performance of transabdominal US examinations; TK, AN and MT: Study conception and design and preparation and evaluation of pathological samples; and SM: Study conception and design and performance of statistical analysis. All authors read and approved the final manuscript.
